# Understanding Health Concerns of Older Adults: Health Personality, Health Activation, and Well-Being

**DOI:** 10.1093/geroni/igaa057.2162

**Published:** 2020-12-16

**Authors:** Peter Martin

## Abstract

Individuals display different levels of concern about their health. These overall concerns may be a result of health personality dispositions based on the five-factor model of personality. They include health neuroticism, health extraversion, health openness, health agreeableness, and health conscientiousness. Furthermore, whether older adults take active care of their health and how they view their overall physical and emotional well-being may depend on these health personality dispositions. This symposium sheds light on the association between health personality, resilience, activation, and well-being. The first presentation provides an overview of our health personality conceptual model and summarizes measurement properties of the Health Personality Assessment. The second presentation highlights demographic differences in health personality. Gender, age, marital status, and regional differences in health personality are reported. The third presentation links health personality with levels of health activation and resilience. Direct and indirect effects of health personality on resilience and health activation are presented. Finally, we highlight results about the relationship of health personality with physical and emotional well-being in later life. All five health personality dispositions directly related to physical and mental health. Our discussion emphasizes practical implications for health practitioners and outlines future research on health personality and outcomes.

